# Hepcidin in neoplastic disease

**Published:** 2013-09-25

**Authors:** CD Nicolae, OA Coman, C Ene, I Nicolae, I Fulga

**Affiliations:** * "Carol Davila" University of Medicine and Pharmacy, Pharmacology and Pharmacotherapy Department, Bucharest; ** "Carol Davila" University of Medicine and Pharmacy, Pharmacology and Pharmacotherapy Department, Bucharest

**Keywords:** hepcidin, iron, cancer, anemia

## Abstract

Abnormalities in iron metabolism are frequent in the neoplastic disease. The relationship between hepcidin and iron homeostasis in cancerous pathology is incompletely known, although it has been studied during the last years. This paper aims to analyze the role of hepcidin in the neoplastic processes, its correlation with carcinogenesis and anemia, and with the disease activity. It must be mentioned that most of the aspects presented need to be verified in practice. Insufficient data are known for showing hepcidin involvement in carcinogenesis, metastasis or in appreciating the response to anemia treatment in neoplasia.

## Introduction

**Fig. 1:**
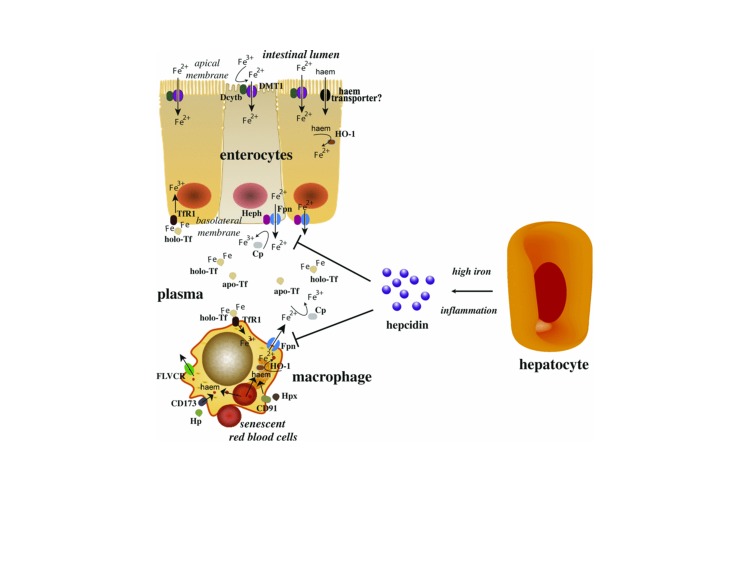
The role of hepcidin in regulating the iron efflux from enterocytes, macrophages and hepatocytes [**6**]

The role of molecular factors that assure iron homeostasis in the body was reviewed after the year 2000, when hepcidin was identified and characterized [**1,2**] as a hormone with a key role in regulating iron metabolism. Hepcidin [**3-7**] has an important role on serum iron, resulting in a decrease of the release of iron from enterocytes and from cells of the reticuloendothelial system. Damage of the regulating mechanism of hepcidin, respectively of modulators that occur in the synthesis of this hormone plays a role in the pathogenesis of diseases such as hemochromatosis, iron deficiency anemia, overload with iron from ineffective erythropoiesis, anemia associated with infection and inflammation, anemia from neoplastic disease. The purpose of this paper focuses on the role of hepcidin in neoplastic processes, its correlation with carcinogenesis and anemia and also with the degree of disease activity. The possibility of hepcidin use in assessing the response to existing therapies in neoplastic disease and the therapeutic intervention over hepcidin represents a current research topic. Data concerning the physiopathology of iron circuit in the body The analysis of data regarding the role of hepcidin in neoplastic pathology first requires a synoptic overview of the factors influencing the iron circuit in the body [**3-10**] (**Fig. 1**).

 The iron absorption from the small intestine is achieved by endocytosis (for heme iron) and by transport proteins and enzymes (for nonheme iron), such as:

 - ferireductase (dCytB), located at the edge of the brush intestinal enterocytes of the intestinal villi, which provides conversion of trivalent iron from food in divalent iron for realizing the transfer in the cells of the intestinal mucosa.

 - DMT1 (Divalent Metals Transporter), localized at the apical membrane of epithelial cells of the intestinal villi and at endosomal vesicles from erythroid cells, which provides the import and the release of iron from endosomes.

 - mobilferrin, a cytoplasmatic transport protein, that realizes the active transfer of iron at the level of basolateral membrane of intestinal cells and of placenta.

 - ferroportin, located in duodenal epithelial cells, macrophages, hepatocytes, embryonic and placental cells, which provides the export of cellular iron in order to be used in erythropoiesis.

 - hephaestin (ferroxidase), located in the basolateral membrane and in vesicles from the intestinal villous enterocytes, which converts the divalent iron to trivalent iron for performing plasmatic transportation.

 - hepcidin, is present in blood, urine and tissues, influences iron absorption and its mobilization depending on erythropoiesis and iron stores.

 The iron transport in plasma is provided by transferrin, a protein of 80 kDa, which contains two high affinity-binding sites for trivalent iron. Lactoferrins existing in milk, saliva, gastric juice, bronchial secretions, and neutrophils, bind iron on mucous and serous surfaces, thus contributing to the antibacterial activity, but they do not supply the reticulocytes with iron. When they reach the plasma lactoferrins they are retained by reticuloendothelial cells.

The iron cells supply is determined by the presence of transferrin receptor TfR1 (transferrin receptor type 1), located in erythroid cells, epithelial cells, macrophages, malignant cells, and TFR2 (transferrin receptor type 2), present in hepatocytes and circulating monocytes, have the ability to bind and internalize the iron bound on transferrin by the phenomenon of endocytosis. DMT1 transports the iron form the endosome in the cytoplasm. Apotransferrin and TfR1 are recycled and transported to the cell surface. HFE protein (gene responsible for hemochromatosis) interacts with TfR1 at the level of cells from intestinal crypts and tissue macrophages.

 Iron storage is realized in ferritin molecules (present in the cytoplasm and lysosomes) and in hemosiderin (stored in the siderosomes from the reticuloendothelial system) in the liver, spleen, bone marrow and muscle fibers. Regulation of iron acquisition by cells and its storage is done through a feedback mechanism at posttranscriptional level, through cytoplasmic proteins, IRF1 and IRF2 (iron-regulatory factors) and IRE (iron responsive element).

Iron excretion in the body is not performed by physiological mechanisms, because there are not specific mechanisms for iron excretion. Iron elimination occurs by cell loss in gastrointestinal, skin, urinary and menstrual cycle in women.

**Hepcidin – synthesis and regulatory factors**

 The year 2000 signified the moment of identifying a hormone with antimicrobial activity LEAP (Liver Expressed Antimicrobial Peptide) [**1**], finding confirmed in 2001 by an independent group of researchers, who call it hepcidin (suggesting the hepatic origin and the bactericidal effect of the molecule) [**2**].

 Hepcidin synthesis. HAMP (hepcidin antimicrobial peptide) gene encoding hepcidin is located on chromosome 19, locus 19q13. The liver is the major organ but not the only one that synthesizes the hepcidin. Other places for synthesis recognized in the body are the heart, kidneys, retina, monocytes, macrophages, spleen cells, alveolar cells, and also pancreatic adipocytes. Hepcidin molecules are present in fluids and tissues of the gestational bag, even from the first quarter of pregnancy [11,12]. In hepatocyte, mature mRNA resulted from gene transcription, assures the development of a prepropeptide of 84 amino acids containing a signal sequence to N-terminal of 24-amino acids, which will be excised and degraded. The molecule of 60 amino acids (positions 25-84) resulting from the enzymatic processing is the prohepcidin, which is secreted in the blood. A cleavage of the prohepcidin, which probably takes place in the blood, between positions 59 and 60, leads to a peptide of 25 amino acids with a C-terminal portion essential for biological activity. Hepcidin-25 is the only one recognized as a mature and active form. Hepcidin-20 and hepcidin-22 were also described and found mainly in the urine, which would be forms of degradation of hepcidin. Hepcidin is distributed in tissues and plasma and excreted by the kidneys.

**Factors modulating the expression of hepcidin**

 Recent data [**13-23**] confirm the involvement of genes that encode proteins HFE (hemochromatosis), HJV (haemojuvelin), TfR2 (transferrin receptor), SLC40A1 (ferroportin) in maintaining iron homeostasis by stimulating the production of hepcidin. Mutations in these genes induce a lower production of hepcidin, a gradual increase in body iron stores and its storage in the organs.

**Fig. 2:**
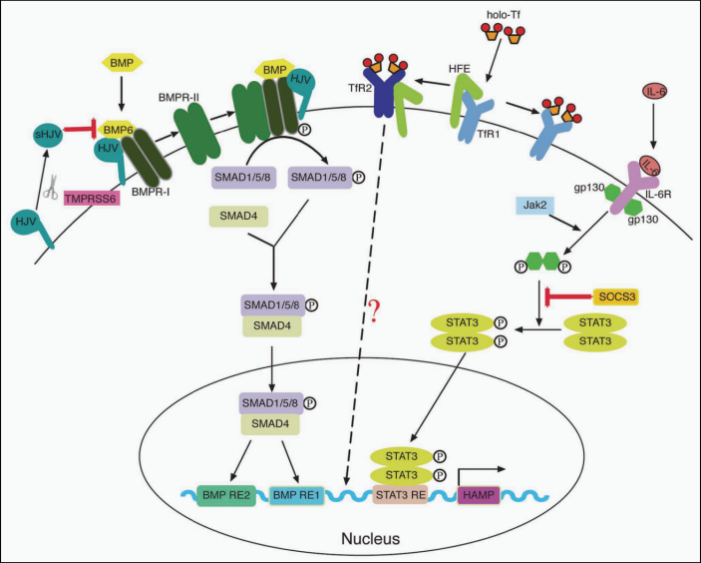
The positive regulation of hepcidin secretion [**7**]

 Hypoxia, anemia, increased erythropoietic activity, decreased iron stores, testosterone, are implicated in the negative regulation of hepcidin. HIF-1 (hypoxia-induced factor) and VHL (von Hippel Lindau tumor suppressor protein) are involved in the regulation of HAMP, both by iron and by hypoxia (**Fig. 2**).

**Fig. 3:**
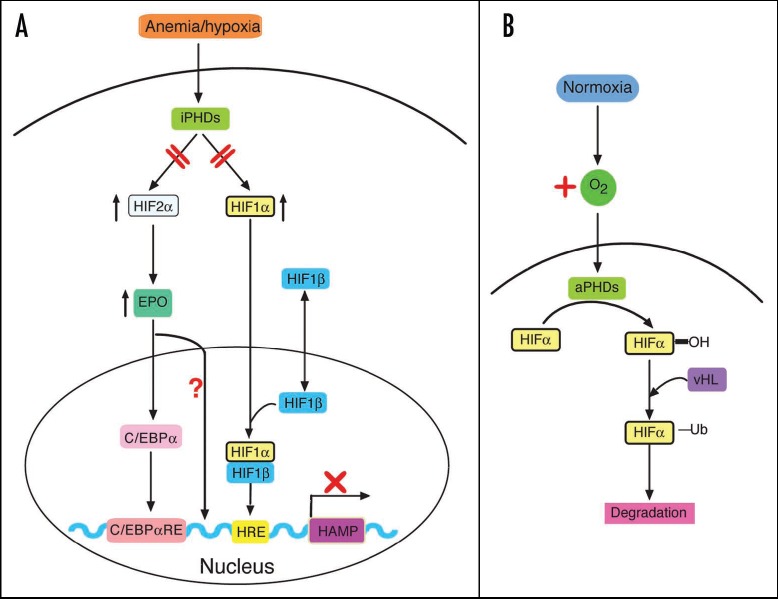
Negative regulation of hepcidin: A) anemia/hypoxia; B) normoxia [**7**]

 The inflammation and the body's iron stores saturation, activates transcription in HAMP at the level of hepatocytes by BMP/SMAD4 (Bone Morphogenetic Proteins), and, respectively, STAT3 (Signal Transducers and Activators of Transcriptions) (**Fig. 3**). Leptine, the substance produced by fatty tissue with cytokine-like action, oncostatin M, free radicals (hydroxyl, superoxide, NO) increase the production of hepcidin.

**Role of hepcidin in the body**

 1. The role of hepcidin in regulating iron levels. Hepcidin must act directly on the source of iron [18,20,21], so, in essence, on three cell types: enterocytes, which absorb dietary iron, macrophages that recycle a large proportion of iron in the body by phagocytosis of erythrocytes at end of their life, and hepatocytes themselves. It was shown that in enterocytes, hepcidin binds to ferroportin, thus preventing the passage of iron from the erythrocyte cytoplasm to the plasma. In macrophages, hepcidin prevents iron export, derived from erythrocytes recycling. In hepatocytes, the hypothesis of iron release is being evaluated.

 2. Antimicrobial action. Hepcidin was identified in the urine as a substance produced by the human body, as part of nonspecific immunity (natural barrier), and, therefore, included in the family of defensins. Its antimicrobial actions were supported by its major role in inducing a decrease in intestinal iron absorption, as well as increased retention of iron in macrophages and liver cells [**2**]. Hepcidin is responsible for the bacteriostatic effect against Gram-positive and Gram negative germs [18,20,21]. Hyperhepcidinmia may be a defense mechanism against bacterial multiplication, which has the ability to use iron directly from transferrin or through their own siderofors. The most significant bactericidal activity of hepcidin was observed against Escherichia coli, Bacillus subtilis, Bacillus megaterium. However, this role of hepcidin is not essential for the body because non-secreting subjects without increased susceptibility to infections were discovered.

 3. Involvement of hepcidin in carcinogenesis and metastasis. Hepcidin, as a key factor in the regulation of iron metabolism, is involved in the pathogenesis of anemia, hemochromatosis, renal failure, and carcinogenesis.

 Many cancers are associated with an increased serum hepcidin such as: breast cancer, colangiocarcinoma, squamous cell carcinoma of the oral cavity, gastrointestinal cancer, non-Hodgkin's lymphoma, brain cancer, small cell lung cancer, mesothelioma, multiple myeloma, ovarian cancer, liver cancer, prostate cancer, kidney cancer, leukemia, multiple myeloma. [**24-35**].

 - Renal cell carcinoma is the most common malignant renal tumor. The presence of much larger quantities of iron was demonstrated in tumor tissue compared to non-tumor tissue [**24-26**]. In addition, iron acts as a cofactor of the catalytic activity of HPHs (HIF prolyl hydroxilases 5) and as a nutrient for tumor cells growth. The involvement of hepcidin (by hepcidin-25 and hepcidin mRNA) in this pathology as a modulator of iron metabolism was suggested. The serum levels of hepcidin-25 and hepcidin mRNA corresponding to hepcidin are much higher for renal cell carcinoma with secondary determinations, compared to that without metastasis. These high levels are markers of an adverse prognosis [**25**]. There was no correlation between serum levels of hepcidin and hepcidin mRNA tissue overexpression. Hepcidin mRNA expression may be considered an independent prognostic factor in renal cancer.

Hepcidin mRNA acts locally at the tumor level, promoting metastatic potential, whereas hepcidin-25 has a systemic action, in response to the progression of renal cell carcinoma. Thus, the serum levels of hepcidin-25 can be used as an indicator of the existence of metastasis, whereas the expression of hepcidin mRNA can be used as a marker of metastatic potential, but it is not associated with a histological differentiation or tumor stage.

 Regarding the role of hepcidin in neoplasms of the bladder and prostate, studies are still ongoing.

 - Colorectal cancer. A growth of the iron levels from the colonocytes can lead to activation of Wnt signaling pathway, which plays a crucial role in colorectal carcinogenesis [**29**]. These assumptions indicate that hepcidin may play a prooncogenic role of internalization and degradation of a cellular protein, which export the iron, ferroportin. The intracellular iron excess from colorectal cancer, most likely due to increased systemic hepcidin values are associated with overproduction of IL-6, STAT-3, TFR2, BMP4, status APC (adenomatous polyposis coli), beta-catenin, p53 [**24,27,29**].

 - Liver cancer. The iron derived from senescent erythrocytes is recycled by macrophages in the spleen or by Kupffer cells in the liver. In macrophages, the iron is released from heme by a reaction catalyzed by hemoxygenase, and then exported and linked to transferrin. Transferrin-iron complex offers the recycled iron to immature red blood cells, and the cycle repeats.

 Iron overload generates reactive oxygen species (ROS), which causes chronic inflammation of the liver. Hepcidin, a key molecule in maintaining iron homeostasis, inhibits the intestinal absorption of iron by enterocytes in the duodenum and iron release by macrophages and hepatocytes [**8,27,28**]. Hepatocellular carcinoma, one of the aggressive forms of liver cancer is a major complication for patients with hemochromatosis, thalasemia, or sideroblastic anemia, because iron is an important nutrient for cancer cells. Most patients with hemochromatosis and cirrhosis develop hepatocellular carcinoma. This fact is important, because cirrhosis plays a major role in hepatocarcinogenesis and is considered a precancerous condition for hepatocellular carcinoma.

 Recent studies have shown that hepcidin mRNA expression is suppressed in hepatocellular carcinoma, regardless of the degree of differentiation of the tumor. Under these conditions, duodenal enterocytes transfer the iron in plasma, which increase the amount of available iron in the body. In addition, inhibition of hepcidin expression is linked also to altered expression of p53, which occurs frequently in patients with hemochromatosis. Hepcidin expression has remained unchanged in non-cancerous tissues, and it is not different in cirrhotic liver from non-cirrhotic one [**24**]. TfR2 (transferrin receptor 2) is a type II transmembrane protein expressed in the liver. An amplification of TfR2 lead to increased hepcidin production. In researches conducted on normal and malignant tissues [**24**], the expression of mRNA for ferroportin-1 and TfR2 tissues does not differ between non-cancerous and cancerous tissues, because hepcidin expression is constantly suppressed in cancerous tissues.

 In the same time, hepcidin-25 was correlated with serum iron and ferritin levels, but not with the level of expression of hepcidin mRNA both in the cancerous tissue and in non-cancerous hepatic tissue.

 Because cancer cells have a high intake of iron, one of the treatment options for liver cancer may be inactivation of the principal importer of iron, TfR1. This can be achieved by monoclonal antibodies anti-TfR1.

 - Brain cancer. The iron is an essential element in neural tissues with a high rate of oxidative metabolism. However, due to the existence of the haemato-encephalic barrier, iron is not in direct contact with the central nervous system. The iron access is regulated by availability of proteins, including TfR1, iron regulatory proteins, ferritin, neogenin, and hepcidin. In the nervous tissue of patients suffering from various neurodegenerative disorders such as Alzheimer's, Parkinson's disease, amyotrophic lateral sclerosis and age-related macular degeneration, there was observed a high amount of iron [**30**]. In addition, neurological tumors such as gliomas have a proportional development with neovascularization. Neovascularization is induced by many factors involving protein von Hippel-Lindau (PVHL) and hypoxia-inducible factor. In normoxia, HIF transcription factor is used for proteosome degradation, as it is stabilized in hypoxic conditions.

 HIF accumulation induces the expression of several proangiogenic proteins, including transferrin and TfR1, which are related to the regulation of cellular iron homeostasis [**30,31**]. Hepcidin (Hamp gene) showed a relatively high level of expression in all brain regions; the highest expression was found in the cortex and anterior thalamus anterior. Hepcidin mRNA produced in the brain tissue may contribute in maintaining iron homeostasis and the protection of central nervous system against infection. Most tumor samples had a reduced hepcidin expression compared with normal cortex, and the values were negligible at the level of astrocytoma’s cell lines. The hypothesis that hepcidin value changes in brain tumors has emerged in comparison with the corresponding normal tissues, according to variation of other parameters such as, HFE, neogenin (NEO1), receptor 1 for transferrin (TFR) and receptor 2 for transferrin (TFR2) [**24,32**].

 4. Hepcidin-biomarker in assessing therapeutic response to epoetin in patients with cancer. Circulating hepcidin in the blood and urine regulates in a negative manner the iron export from enterocytes, macrophages, hepatocytes, by its binding to the ferroportin, internalization and degradation of a membrane channel. Decreased iron efflux from deposit tissues to plasma and reduction of intestinal absorption contribute to the onset anemia in neoplastic disease. In the context of cancer, anemia is exacerbated by chemotherapy.

 Increased incidence of anemia in patients with cancer has generated numerous experimental studies that have sought to quantify hepcidinmia and hepcidinuria in patients with such pathologies [**2,3,18,20**]. In patients with inflammatory anemia, a positive correlation between serum hepcidin and the presence of an inflammatory process (IL 6, C-reactive protein) was revealed. Hepcidin-25 level does not correlate with the severity of anemia in patients with colorectal cancer, which confirms the multifactorial genesis of anemia in patients with malignancy.

 Clinical and laboratory evidence [**36,40**] suggests a possible use of hepcidin as a predictive marker for the response to the treatment with epoetin in patients with solid tumors (lung cancer, multiple myeloma, non-Hodgkin's lymphoma, mixed tumors). Retrospective analysis of five different studies that had as main objective the correction of anemia, showed that injection of epoetin reduce hepcidin expression and facilitates intestinal absorption of iron, approximately 35-40% of cancer patients are resistant to treatment with epoetin, patients low baseline hepcidin responded better to treatment with epoetin, at higher values of initial hepcidin the number of non-responders is higher.

## Conclusions

1. Hepcidin is a protein mainly synthesized in the liver, which has a regulatory effect on the iron metabolism in the body.

 2. Multiple studies brought proofs about the implication of hepcidin in carcinogenesis and also in the metastatic processes. Hepcidin acts directly as prooncogene or indirectly through iron. Iron could be implicated in the processes of carcinogenesis from rectum, bowel, liver and prostate by generation of reactive oxygen species (ROS), with a proinflammatory effect. 

 3. Hepcidin may represent the cause of an inadequate response to the treatment with agents that stimulate erythropoiesis in patients with various forms of cancer.

 4. The therapeutic intervention on hepcidin could be a solution in the treatment of patients with anemia associated with cancer.
